# Reduction of malaria prevalence after introduction of artemisinin-combination-therapy in Mbeya Region, Tanzania: results from a cohort study with 6773 participants

**DOI:** 10.1186/s12936-018-2389-z

**Published:** 2018-06-26

**Authors:** Guenter Froeschl, Elmar Saathoff, Inge Kroidl, Nicole Berens-Riha, Petra Clowes, Leonard Maboko, Weston Assisya, Wolfram Mwalongo, Martina Gerhardt, Elias Nyanda Ntinginya, Michael Hoelscher

**Affiliations:** 1 Division of Infectious Diseases and Tropical Medicine, University Hospital, LMU Munich, Leopoldstr. 5, 80802 Munich, Germany; 2 German Center for Infection Research (DZIF), Partner Site Munich, Leopoldstr. 5, 80802 Munich, Germany; 3 National Institute of Medical Research-Mbeya Medical Research Centre, P.O. Box 2410, Hospital Hill Road, Mbeya, Tanzania

**Keywords:** Artemisinin-based combination therapy, Cohort study, Normalized Difference Vegetation Index, Bed net, Malaria, *Plasmodium falciparum*, Prevalence, Rapid diagnostic test, Mbeya Region, Tanzania

## Abstract

**Background:**

A marked decline in malaria morbidity and mortality has been reported after the introduction of artemisinin-based combination therapy (ACT) in high malaria prevalence countries in Africa. Data on the impact of ACT and on the prevalence of malaria has so far been scarce for Southwest Tanzania.

**Methods:**

Between 2005 and 2011, a large general population cohort in the Mbeya Region in the south-west of Tanzania has been surveyed within the EMINI-study (Evaluation and Monitoring of the Impact of New Interventions). Participants were examined once per year, including rapid diagnostic testing for malaria. ACT was introduced in the region according to national guidelines in the time period 2006/2007, replacing sulfadoxine/pyrimethamine as first-line therapy. In four study sites, 6773 individuals who participated in the first two of three consecutive survey visits in the period from 2006 to 2009 were included in this analysis. The prevalence of *Plasmodium* infection prior to and after the introduction of ACT was compared by logistic regression, with consideration of climatic variability, age, sex, socio-economic status and bed net use as potential confounders.

**Results:**

A significant reduction over time in the prevalence of *Plasmodium falciparum* infection from 2.5 to 0.3% was shown across the four study sites. The decline was not explained by other factors included in the analysis, therefore, the decline over time most likely reflects the impact of introduction of ACT in the study area.

**Conclusions:**

The longitudinal study showed a significant and relevant decline in the prevalence of *P. falciparum* infection after introduction of ACT, which could not be explained by potential confounders. The data suggests that artemisinin-based combinations are not only an effective instrument for reduction of immediate morbidity and mortality, but also for reduction of transmission rates.

## Background

According to the World Malaria Report 2016, to date still more than 210 million persons suffer from malaria infections every year, with 429,000 malaria deaths in 2015 worldwide. The major burden of malaria still lies within Africa with more than 190 million cases and 394,000 deaths annually. Almost all malaria cases in sub-Saharan Africa are caused by the species *Plasmodium falciparum*. This high mortality in Africa is aggravated by the high proportion of deaths in the under-5 age-group, which account for about 90% of all malaria related deaths, more than in any other global region.

The East-African country Tanzania has a total population of 45 million, of whom 44% are under the age of 15, and 15% under the age of 5 years. A Tanzanian has a combined life expectancy at birth of 56 years, the total fertility rate is 6.3 children per woman, the annual population growth rate is 2.7% [1]. The per capita gross domestic product is US$ 652 and the Human Development Index of 0.521 is ranking 151st out of 186 countries [2]. According to World Health Organization (WHO) data on Tanzania, malaria causes 10% of all deaths in the under-5-age-group, while the overall under-5-mortality in 2012 was 52 deaths per 1000 live births [3]. The total malaria deaths for Tanzania for all age groups was more than 20,000 in 2012, and it may be assumed that the large majority of these deaths are again found in children below 5 years. In a prevalence study conducted in Central Tanzania in 2015, 22.5% of participants between 1 and 10 years of age and 26.8% between 11 and 20 years were found rapid diagnostic test (RDT) positive for malaria [4]. Malaria still is a major public health concern in Tanzania and among the top five causes of death for children under 5 years of age [3].

In order to tackle this problem, particularly in countries with a high malaria burden, which are at the same time low-resource-countries, global stakeholders, such as the WHO, the Global Fund to Fight AIDS, Tuberculosis and Malaria and the Gates Foundation have engaged in promoting aims of malaria reduction that have been set forth in the Millennium Development Goals. Consecutively the World Health Assembly and the Roll Back Malaria initiative have envisaged a reduction by 75% in malaria disease burden between the years 2000 and 2015. Interventions include vector and exposure control through indoor residual spraying (IRS), distribution of insecticide-treated bed nets (ITN), introduction of RDT for differentiation between true malaria cases and non-malarial febrile illnesses, and introduction of artemisinin-based combination therapy (ACT) as first-line treatment [5]. Subsequently, numerous high malaria burden countries such as Zambia or Tanzania have reported declines in malaria incidence [6–8]. However, data on malaria prevalence remains patchy, and solid monitoring mechanisms are absent.

Even after the large-scale implementation of rapid diagnostic testing and ACT in Tanzania, high prevalences of parasitaemia in many regions imply a persistent risk of transmission to vulnerable populations, such as infants. In a study from Northern Tanzania in 2012/2013, thus well after the introduction of ACT and RDTs, a prevalence of plasmodium infection of 12.8 and 11.0% were detected in pregnant women and in infants respectively, regardless of presence of symptoms [9]. Another study from Northern Tanzania that was trying to identify Dengue and Chikungunya virus infections as causes of febrile illness employed the WHO case definition criteria for Dengue and Chikungunya as criteria for recruitment of febrile patients. Still 8.8% of these participants were infected with malaria [10]. The adequate use of anti-malarials, or even an anti-malarial stewardship, as the key factor for prevention of drug resistance remains a challenge, while uncontrolled distribution of valuable treatment options, such as ACT remains common practice [11].

In this study, the prevalence of malaria RDT positivity before and after the introduction of ACT was compared in order to estimate the effect of ACT introduction on malaria in the study population.

## Methods

### Study setting, study design and population

The Mbeya Region with a population of 2.7 million is located in the Southwest of Tanzania, a highland area with mountains ranging up to 3000 m in altitude [1]. The lowest point is the shore of Lake Malawi at about 500 m above sea level. The climate at lower elevations is subtropical with a rainy season from December to April. The average annual rainfall for Mbeya (years 1991–2015) is 883 mm [12]. Night-time temperatures in the dry season can drop below 10 °C at higher altitudes.

This study is an observational, longitudinal and closed cohort study, based on the EMINI (Establishment of the infrastructure for the Evaluation and Monitoring of the Impact of New Interventions) cohort, established since May 2006 by the Tanzanian National Institute of Medical Research-Mbeya Medical Research Centre (NIMR-MMRC) in collaboration with the Division of Infectious Diseases and Tropical Medicine, University Hospital, LMU Munich. The design follows a pre- and post-intervention structure, where the intervention was a switch of government first-line malaria treatment from SP to ACT in the whole population. In order to properly represent the regional diversity in terms of geography, urbanization and living conditions, nine study sites in the Mbeya Region were purposively selected for participation in the EMINI cohort, covering characteristics, such as urbanization and different levels of altitude. In preparation for EMINI, a complete population census was performed in all nine sites. In order to get a geographically balanced sample and avoid random spatial clustering, each site was then subdivided into 6–10 areas, from each of which 10% of households were randomly selected for participation in the EMINI cohort. All household members of all ages from these selected households were then invited for participation, resulting in a cohort of more than 18,000 individuals in the nine EMINI sites. Because the introduction of ACT coincided with the set-up of the cohort in five of the nine sites, no consistent pre-ACT baseline data is available for these sites. Hence, only the remaining four sites, where ACT was introduced after completion of the first baseline visit were included in this analysis, comprising 7957 individuals. Out of these, 1184 individuals were not present at the second household visit, resulting in 6773 individuals who were included in our analyses.

**Fig. 1:**
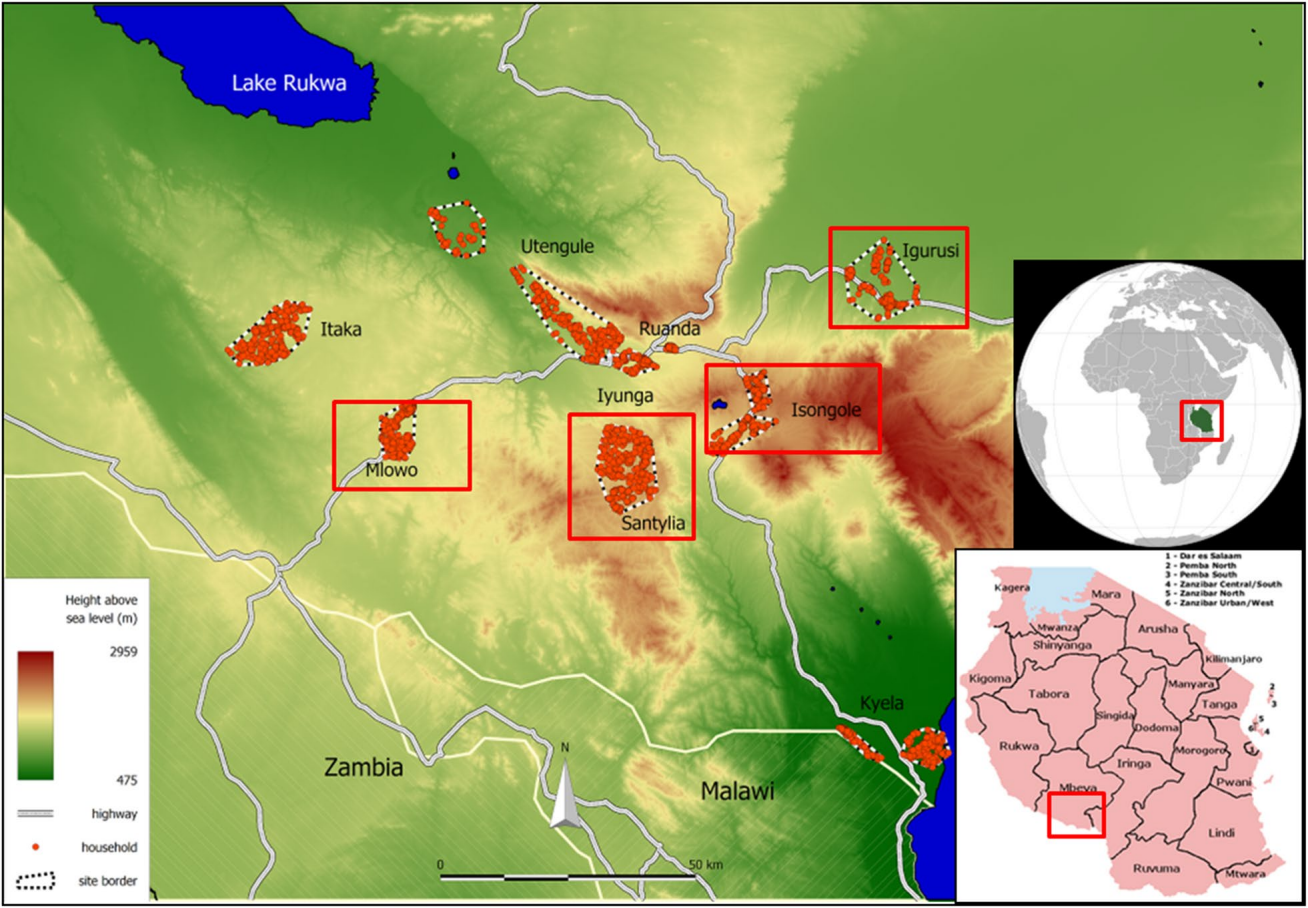
Map of study sites in the Mbeya Region. The larger image shows the location of the nine EMINI study sites in the Mbeya Region with red dots indicating household positions. Red rectangles indicate the four sites included in our analysis. The inserted images show the locations of Mbeya Region in South-West Tanzania, and of Tanzania in East Africa

The included four sites are the rural areas of Igurusi (1190 m, population 19,796), Mlowo (1590 m, population 21,307), Santylia (2020 m, population 21,149) and Isongole (2040 m, population 20,360) (Fig. 1). The above reference population numbers were retrieved from an initial population census before the start of the actual EMINI cohort study, while the indicated altitudes of the sites are the mean altitudes of household positions in each site, which were retrieved from the global elevation model (version 2.1) of the US National Aeronautics and Space Administration (NASA) Shuttle Radar Topography Mission (SRTM) [13, 14].

The analysis includes data from three annual rounds of household visits which took place between May 2006 and June 2007 (visit 1), July 2007 and July 2008 (visit 2), and July 2008 and June 2009 (visit 3). During each round, the same sites and households were visited at about the same time of the year.

### Variables and instruments

*Plasmodium falciparum* infection was tested using the ICT Malaria Combo Cassette Test (ICT Diagnostics, Cape Town, South Africa). This RDT is an immunochromatography test with antibodies against histidine-rich protein 2 (HRP2), which is species-specific for *P. falciparum*, in combination with antibodies against aldolase which is detected in *P. falciparum*, *Plasmodium malariae*, *Plasmodium ovale* and *Plasmodium vivax* infections. For this study *P. falciparum* positivity was defined as any RDT result with both a positive control line and a positive HRP2 line, regardless of the result of the aldolase line. The test was performed on every participant at each household visit, regardless of clinical signs of malaria. Participants with a positive result were offered immediate treatment with ACT. Since ACT was introduced in the entire region, no control group continuing to be exposed to the previous first-line treatment could be included in the analysis. RDTs and immediate treatment provision, restricted to confirmed cases only, were already in place in all sites prior to the study initiation.

Information on personal and household characteristics (age, sex, and indicators of socio-economic status) was collected using questionnaire based face-to-face interviews, which were held in Kiswahili. Bed net ownership and housing quality was ascertained by visual inspection. A socio-economic status (SES) score was constructed as a composite and standardized variable based on a principal component analysis of several household assets and housing quality [15, 16].

The geographical position of each participating household was determined using handheld GPS (Global Positioning System) receivers. Vegetation characteristics were quantified using annual averages of the Normalized Difference Vegetation Index (NDVI), which is based on satellite measurements as provided by the US National Aeronautics and Space Administration (NASA). Differences in light absorption spectra measured by spectral cameras in satellites allow the assessment of vegetation density, which in turn allows conclusions on previous floral growth conditions, such as humidity and rainfall profile. This vegetation data is available from the NASA Moderate-Resolution Imaging Spectroradiometer (MODIS) Terra Mission [17, 18]. NASA gives as an approximate interpretation a range for the NDVI from 0.2 to 0.3 for shrub and grassland, while values between 0.6 and 0.8 indicate temperate and tropical rainforests. Elevation data were retrieved from the NASA Shuttle Radar Topography Mission (SRTM) global digital elevation model, version 2.1 [13]. NDVI and elevation data were averaged for a buffer area of 1000 m radius around each household position to characterize the situation at household level.

The performed analyses are based on data before (visit 1) and after (visit 2) introduction of ACT. In order to corroborate the sustainability of reduction in *P. falciparum* infections, prevalences for visit 3 are also reported.

### Statistical analysis

All interview and medical examination data were collected using hand-held personal digital assistance devices and then imported into a Microsoft Access (Microsoft Corporation, Redmond, USA) database. Laboratory results were documented on paper forms, double entered into a Microsoft Access database and compared and corrected for data entry errors. Data analysis was performed using Stata version 14.2 (StataCorp, College Station, TX, USA). The univariable description of the cohort uses proportions to summarize categorical variables. Continuous variables are summarized by their median and interquartile range, since none of them was normally distributed according to the Shapiro–Francia test.

Two mixed effects multivariable logistic regression models with random effects for study site and household to adjust for clustering were constructed. The first model examines the effects of various factors on the baseline prevalence of malaria RDT positivity, with the RDT result of visit 1 as the outcome and sex, age, bed net ownership, SES and NDVI as covariates. The second model targets the change in RDT positivity from visit 1 to visit 2. Change is dichotomously defined as a change from positive in visit 1 to negative in visit 2 versus all other scenarios combined (positive at both visits, negative at both visits, negative in visit 1 to positive in visit 2). In this model the independent variables sex, age, change in bed net ownership, change in SES and change in NDVI from visit 1 to visit 2 were included. In 4 out of 6773 participants data on SES was missing, therefore the models were run on 6769 participants.

### Ethical considerations

This study follows the principles of the Helsinki Declaration. All participants had signed an informed consent form prior to inclusion. For all minors below the age of 18 years their legal guardians signed the informed consent form and children between 12 and 18 years additionally signed an assent form. Ethics approval of the cohort study within the EMINI framework was obtained from the Tanzanian National Institute for Medical Research.

## Results

### Cohort background characteristics

The lower lying sites of Igurusi and Mlowo are characterized by lower NDVI values than Santylia and Isongole. The numbers of inhabitants of the four included sites according to the EMINI census data, the mean altitude and the mean vegetation-index of households at the respective sites are detailed in Table 1.

**Table 1:** Study site characteristics

Site	Mean altitude (m)	Total population	Number of participants	Mean NDVI at visit 1	Mean NDVI change visit 1 to visit 2 (%)
Igurusi	1190	19,796	1126	0.45	− 0.5
Mlowo	1590	21,307	1956	0.45	− 2.5
Santylia	2020	21,149	1802	0.57	− 0.2
Isongole	2040	20,360	1889	0.57	+ 2.9

Table 2 describes the study population of the four sites. Female participants were significantly older than male participants (median 17.4 years for females, 14.4 years for males, Mann–Whitney p < 0.0001). In Igurusi and Mlowo average SES was higher than in Santylia and Isongole.

**Table 2:** Study population characteristics

Site	Igurusi	Mlowo	Santylia	Isongole	Total
Number of participants	1126	1956	1802	1889	6773
Proportion of females; n/N (%)	593/1126 (0.53)	1027/1956 (0.53)	1001/1802 (0.56)	1045/1889 (0.55)	3666/6773 (0.54)
Median age at visit 1 in years (IQR)	16.5 (8.3; 36.1)	14.9 (7.8; 33.1)	16.0 (7.3; 34.2)	16.0 (7.8; 3.4)	15.7 (7.6; 34.7)
Bed net ownership; n/N (%)					
Visit 1	1030/1126 (91.5%)	646/1956 (33.0%)	0/1802 (0.0%)	4/1889 (0.2%)	1680/6773 (24.8%)
Visit 2	998/1126 (88.6%)	796/1956 (40.7%)	32/1802 (1.8%)	31/1889 (1.6%)	1857/6773 (27.4%)
Visit 3	1022/1083 (94.4%)	1033/1845 (56.0%)	0/1622 (0.0%)	27/1762 (1.5%)	1082/6312 (33.0%)
Change in bed net ownership (visit 1 to visit 2)	− 3.1%	+ 23.2%	NA	+ 681%	+ 10.5%
Median SES-score (IQR)					
Visit 1	0.01 (− 0.49; 0.64)	0.13 (− 0.23; 0.63)	− 0.42 (− 0.75; − 0.06)	− 0.36 (− 0.73; 0.00)	− 0.17 (− 0.58; 0.23)
Visit 2	− 0.02 (− 0.44; 0.54)	0.16 (− 0.25; 0.71)	− 0.52 (− 0.82; − 0.14)	− 0.42 (− 0.73; 0.14)	− 0.21 (− 0.62; 0.32)
Visit 3	0.06 (− 0.54; 0.61)	0.37 (− 0.13; 0.85)	− 0.51 (− 0.84; − 0.02)	− 0.37 (− 0.80; 0.20)	− 0.14 (− 0.66; 0.45)
*P. falciparum* RDT positivity; n/N (%)					
Visit 1	82/1126 (7.3%)	60/1956 (3.1%)	7/1802 (0.4%)	17/1889 (0.9%)	166/6773 (2.5%)
Visit 2	4/1126 (0.4%)	9/1956 (0.5%)	3/1802 (0.2%)	11/1889 (0.6%)	27/6773 (0.4%)
Visit 3	9/980 (0.9%)	4/1670 (0.2%)	1/1447 (0.1%)	0/1479 (0.0%)	14/5576 (0.3%)
Change in RDT + (visit 1 to visit 2) (%)	− 95.1	− 85.0	− 56.4	− 35.6	− 83.7

### Vegetation index

The four sites showed very different NDVI distributions, which corresponds to the varying physiognomy of the locations. Most NDVI values were in the range between 0.3 and 0.6, and thus between grassland and temperate/tropical forest according to NASA [19]. This reflects the savannah landscape that is mostly found in the populated areas of Mbeya Region. Mean relative NDVI change between visit 1 and visit 2 for each site ranged from − 2.55 to + 2.90% (Table 1). While NDVI decreased in Igurusi (− 0.50%, 95% confidence interval: − 0.61 to − 0.39%), Mlowo (− 2.55, − 2.60 to − 2.49%) and Santylia (− 0.21, − 0.44 to 0.01%), Isongole showed an increase in NDVI (2.90; 2.71 to 3.09%). However, overall these changes can be considered as minimal.

### *Plasmodium falciparum* prevalence

**Fig. 2:**
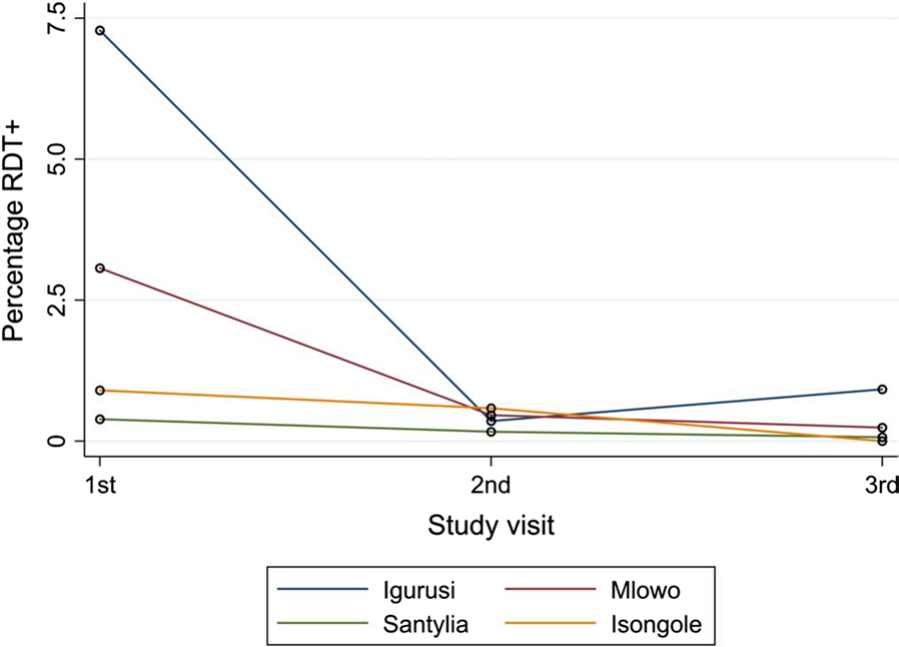
Prevalence of *P. falciparum* positive RDT results over time by study site. ACT was introduced between visit 1 and visit 2 in each study site

Marked differences between the different study sites regarding baseline prevalences of *P. falciparum* infection were found (Table 2). As expected, lower prevalences already at baseline could be shown for the two locations at higher altitudes (0.4% in Santylia and 0.9% in Isongole) compared to the lower lying sites (7.3% in Igurusi and 3.1% in Mlowo). For all four included study sites, a decrease of *P. falciparum* infection prevalence after introduction of ACT was observed regardless of altitude, which most prominently occurred between the baseline visit and the first follow-up visit. Figure 2 highlights the crude decline of prevalence of positive RDT results for all sites over time.

**Fig. 3:**
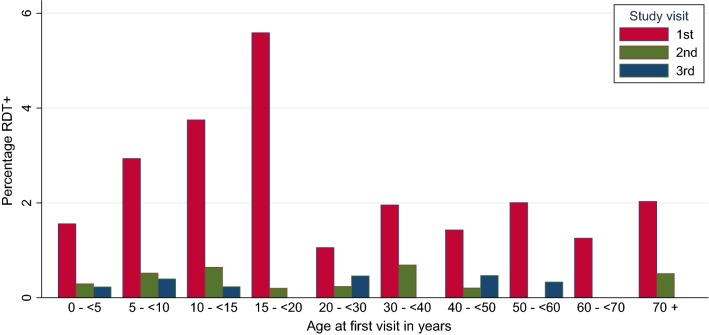
Prevalence of *P. falciparum* RDT positivity over time by age group. Malaria RDT positivity for *P. falciparum* before (visit 1) and after (visits 2 and 3) ACT introduction combining all data from all four study sites

Across all four included sites, 160 out of 6773 individuals were RDT positive at visit 1 and RDT negative at visit 2; six individuals were RDT positive at both visit 1 and 2; and 21 individuals were RDT negative at visit 1 and turned positive at visit 2; all remaining individuals were RDT negative at both visit 1 and visit 2. At baseline, participants between 10 and 20 years of age were more often *P. falciparum* positive (4.3%; 69/1594) than all other age groups (Fig. 3). Furthermore, males across all age groups below 40 years had a higher prevalence of *P. falciparum* positivity (3.0%; 75/2525) than females of the same age group (2.4%; 70/2973).

**Fig. 4:**
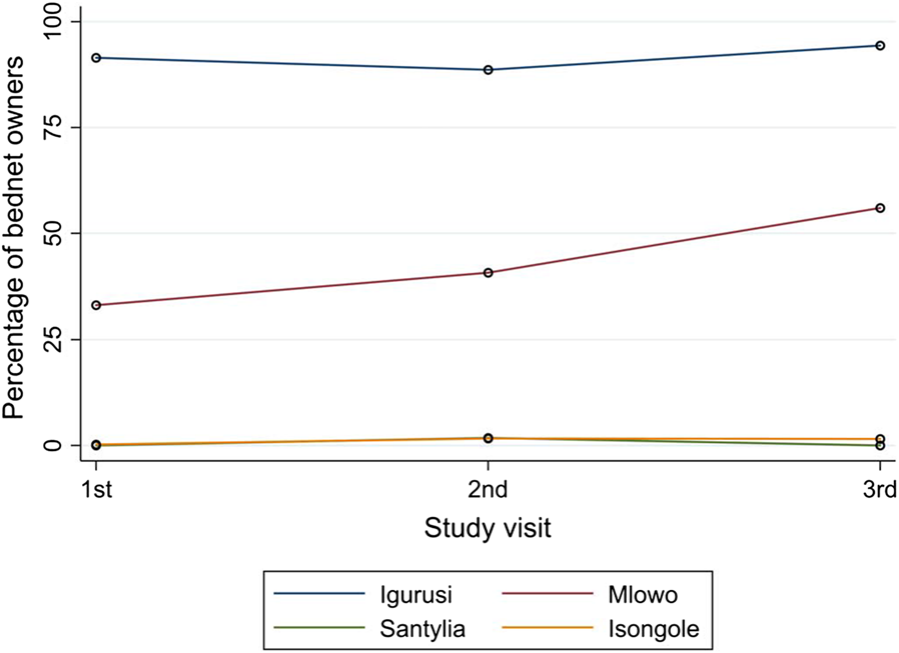
Proportions of bed net owners over time

The proportion of bed net owners corresponded to the prevalence of malaria in the sites, with more bed net owners in the lower lying sites where malaria infection is common. As for the malaria high-prevalence site Igurusi, where also the proportion of bed net owners was high, individuals from households with bed nets showed lower prevalences of parasitaemia compared to individuals from households without bed nets located in the same site (Table 3). Figure 4 shows the proportion of bed net owners by site for all four sites over the three survey periods.

**Table 3:** Change in bednet ownership and *P. falciparum* RDT positivity at visits 1 and 2 in Igurusi site

Change in bed net ownership	RDT + at visit 1 n/N (%)	RDT + at visit 2 n/N (%)
Lost bed net from visit 1 to visit 2	5/77 (6.5%)	0/77 (0.0%)
No bed net at both visits 1 and 2	15/51 (29.4%)	0/51 (0.0%)
Bed net at both visits 1 and 2	58/953 (6.1%)	4/953 (0.4%)
Acquired bed net from visit 1 to visit 2	4/45 (8.9%)	0/45 (0.0%)

This comparison of change in bed net ownership and *P. falciparum* infection is provided for Igurusi only, the location with the highest overall prevalence of *P. falciparum* infection at visit 1

Change in bed net ownership between visits 1 and 2 and its potential influence on change in malaria prevalence was further analysed for Igurusi, the site with the highest malaria prevalence. Table 3 shows, that *P. falciparum* RDT positivity dropped substantially in all four groups, including participants who owned a bed net at the first visit but had lost it before the second visit.

### Multivariable analysis

In total 6769 participants with *P. falciparum* RDT results for the first and second visit were included in the two uni- and multivariable mixed effects logistic regression models. The first model aims to identify factors that might influence *P. falciparum* infection at the first visit. The second model examines the influence that potential change of the above identified factors between the visits might have on the decline in malaria between visits. The first model revealed significantly higher odds of *P. falciparum* infection for participants between 5 and 10 (OR 2.05, 95% CI 1.32–3.17) and between 10 and 20 years of age (OR = 3.04, 95% CI 2.14–4.32) compared to children below 5 years. Bed net ownership, and SES were negatively associated with RDT positivity whereas NDVI showed a positive association. These associations were relatively strong, but significant only in univariable, and not in multivariable analysis (Table 4).

**Table 4:** Uni- and multivariable mixed effects logistic regression model for *P. falciparum* RDT positivity at visit 1

Covariate	N	N-pos.	%-pos.	Univariable			Multivariable		
				OR	95% CI	*p* value	OR	95% CI	p value
Gender									
Female^a^	3663	84	2.29	1.00	– to –	–	1.00	– to –	–
Male	3106	82	2.64	1.14	(0.99 to 1.31)	0.0747	1.10	(0.95 to 1.26)	0.1929
Age at Srv1 (years)									
0– < 5^a^	1026	16	1.56	1.00	– to –	–	1.00	– to –	–
5– < 10	1158	34	2.94	2.07	(1.35 to 3.17)	0.0009	2.05	(1.32 to 3.17)	0.0013
10– < 20	1592	69	4.33	3.10	(2.20 to 4.37)	< 0.0001	3.04	(2.14 to 4.32)	< 0.0001
20– < 40	1720	26	1.51	1.03	(0.49 to 2.18)	0.9356	1.06	(0.50 to 2.22)	0.8802
≥ 40	1273	21	1.65	1.09	(0.48 to 2.43)	0.8426	0.99	(0.42 to 2.32)	0.9808
Bed net owned									
No^a^	5089	89	1.75	1.00	– to –	–	1.00	– to –	–
Yes	1680	77	4.58	0.44	(0.22 to 0.92)	0.0281	0.56	(0.18 to 1.73)	0.3139
SES-score									
Per unit	–	–	–	0.72	(0.64 to 0.81)	< 0.0001	0.81	(0.65 to 1.00)	0.0515
NDVI									
Per 0.1 NDVI units	–	–	–	2.20	(1.01 to 4.82)	0.0479	1.83	(0.81 to 4.17)	0.1490

Using robust variance estimates and including site and household as random effects to adjust for clustering, N = 6769

*N* number of observations, *N-pos.* number of positives, *%-pos.* percent positive, *OR* odds ratio, *95% CI* 95% confidence interval

^a^ Reference stratum

Nevertheless, these factors were included into the second model, where the influence of changes in bed net ownership, NDVI and SES on the observed change in malaria prevalence between the first and second visit were assessed, again using uni- and multivariable mixed effects logistic regression models, but this time with loss of *P. falciparum* infection, i.e. change in RDT status from positive to negative as the outcome.

Although unchanged between visits, age at visit 1 and gender were included into the models for loss of *P. falciparum* infection as potential confounders (Table 5). The multivariable model showed significantly higher odds of changing to negative for those groups who also had higher odds of infection at visit 1: male participants and participants between 5 and 10 (OR = 1.94, 95% CI 1.29–2.91) and between 10 and 20 years of age (OR = 2.98, 95% CI 2.10–4.23) compared to children below 5 years, had higher odds of changing to *P. falciparum* negative. Participants who lost or obtained a bed net between surveys both changed more often to negative than participants with unchanged bed net status (3.79 and 2.84% versus 2.29% respectively). However, when adjusting for site and household in the mixed effects models, the odds of losing infection were considerably lower for participants who lost their bed net compared to those with unchanged bed net ownership in both the uni- (OR = 0.74, 95% CI 0.67–0.82) and multivariable analysis (OR = 0.76, 95% CI 0.57–1.02), whereas the odds for those who obtained a bed net were nearly the same as for those where bed net ownership did not change.

**Table 5:** Uni- and multivariable mixed effects logistic regression models to assess the potential influence of changes in bed net ownership, SES and NDVI between visit 1 and visit 2 on change of *P. falciparum* RDT result from positive at visit 1 to negative at visit 2

Covariate	N	N-chgd.	%-chgd.	Univariable			Multivariable		
				OR	95% CI	p value	OR	95% CI	p value
Gender									
Female^a^	3663	80	2.18	1.00	– to –	–	1.00	– to –	–
Male	3106	80	2.58	1.17	(0.97 to 1.41)	0.0950	1.12	(0.94 to 1.35)	0.2042
Age at visit 1, years									
0– < 5^a^	1026	16	1.56	1.00	– to –	–	1.00	– to –	–
5– < 10	1158	32	2.76	1.95	(1.31 to 2.88)	0.0009	1.94	(1.29 to 2.91)	0.0013
10– < 20	1592	67	4.21	3.00	(2.12 to 4.26)	< 0 .0001	2.98	(2.10 to 4.23)	< 0.0001
20– < 40	1720	25	1.45	0.98	(0.49 to 1.99)	0.9658	0.99	(0.47 to 2.07)	0.9823
≥ 40	1273	20	1.57	1.02	(0.50 to 2.07)	0.9588	1.00	(0.49 to 2.03)	0.9947
Change in bed net ownership									
Lost net	211	8	3.79	0.74	(0.67 to 0.82)	< 0.0001	0.76	(0.57 to 1.02)	0.0669
No change^a^	6170	141	2.29	1.00	– to –	–	1.00	– to –	–
Received net	388	11	2.84	0.99	(0.49 to 1.98)	0.9732	1.04	(0.48 to 2.24)	0.9209
Change in SES score									
(Per unit)	–	–	–	0.93	(0.74 to 1.17)	0.5614	0.96	(0.70 to 1.31)	0.7984
Relative change in NDVI									
Per 1% change	–	–	–	1.10	(1.00 to 1.21)	0.0565	1.10	(0.99 to 1.22)	0.0756

Using robust variance estimates and including site and household as random effects to adjust for clustering, N = 6769

*N* number of observations, *N-chgd. and %-chgd.* number and percentage of participants who changed from *P. falciparum* positive at visit 1 to negative at visit 2, *OR* odds ratio, *95% CI* 95% confidence interval

^a^ Reference stratum

Change in SES showed a very weak and non-significant negative association with loss of malaria infection. Change in NDVI showed a stronger but non-significant positive association, in both the uni- and multivariable analysis (OR = 1.10, 95% CI 0.99–1.22), meaning that loss of *P. falciparum* infection between visits was higher when NDVI increased, which is counterintuitive.

## Discussion

In the EMINI cohort, replacement of sulfadoxine/pyrimethamine (SP) by ACT as first-line therapy in the years 2006 and 2007 resulted in a significant and relevant reduction of *P. falciparum* prevalence. In this study, malaria status was determined by RDT, which reflects common procedures in health care systems in malaria endemic countries. This strategy is somewhat hampered by limited sensitivity and specificity, which may be particularly challenging the positive predictive value of any given RDT in a setting where malaria prevalence has been already substantially reduced. Between baseline (visit 1) and the first follow up (visit 2) an overall 83.7% reduction in *P. falciparum* prevalence could be shown. The effect was sustained and even consolidated afterwards, resulting in an overall prevalence of *P. falciparum* RDT positivity of only 0.3% at visit 3 compared to 2.5% at baseline. Similarly promising findings have been reported from other countries that are endemic for malaria [6]. A reduced prevalence of parasitaemia is a key factor in reducing transmission, in particular the reduced prevalence of gametocyte carriers, which is achieved through the gametocidal activity of ACT. However, some studies still suggest high prevalences of Plasmodium infection in the general population after ACT implementation as a continued driver for sustained transmission [20–22].

The crude prevalence of Plasmodium infection differed significantly between sites, with higher prevalences in the lower altitude sites of Igurusi and Mlowo, compared to the high altitude sites Santylia and Isongole. The mixed effects logistic regression model found that age between 5 and 20 years was a significant predictor for a higher risk of *P. falciparum* infection, an age dependent distribution of prevalence, which has been described in other studies [4, 6]. It appears that younger children are better protected, for example by the use of the existing bed nets, whereas adolescents in the age range from 10 to 20 years seem to be particularly exposed due to staying outside homes also during evening and night hours with greatest risk of transmission. At baseline, males were slightly more often *P. falciparum* infected than females, as has been shown in other studies [4]. The model further showed that owning a bed net and SES are negatively, and NDVI values at the place of residence are positively associated with *P. falciparum* infection, although these associations were significant only in univariable analysis (Table 4).

When looking at the change of *P. falciparum* infection status from visit 1 to visit 2, we found that age between 5 and 20 years was the only factor that was significantly associated with a change from *P. falciparum* positive to negative between the two visits. Since this age group had the highest malaria prevalence at baseline, the potential for changing to malaria negative is also highest, which is corroborated by our findings. The same is true for the non-significant association of male gender with loss of malaria infection. Changing SES only showed a weak association with loss of malaria infection.

The odds for switching to RDT negative for participants who obtained a bed net between surveys and for those whose bed net ownership status did not change are very similar. The large majority of individuals had a stable bed net ownership status, that is: keeping their bed net or staying without one, which in turn depended on the background prevalence of *P. falciparum* infection. The group that received a bed net remained small, only 11 individuals in this group showed a switch from infection status positive at visit 1 to negative at visit 2 (Table 5). This shows that a higher bed net coverage is not the driving factor for the observed drop in *P. falciparum* infection after ACT introduction, at least in this setting with the reported baseline coverage of bed nets. It has to be pointed out, that the variable of bed net ownership bears its limitations, as actual use of the bed net is not assessed.

The association of increasing NDVI with switching to malaria negative, which is close to significant, is counterintuitive. Increasing NDVI, as a marker of increasing humidity, should rather have been associated with a lower decrease in malaria infection. Thus this result is hard to explain, but at least shows that changes in vegetation do not explain the drop of malaria prevalence between visit 1 and visit 2. It has to be pointed out, that on a site level the differences in altitude between the sites will have played a greater role than NDVI. However, NDVI values were determined on a household level, and here neither significant association nor intuitive trend could be established.

A challenge in malaria control may be seen in areas with inherently rather low malaria prevalence, such as the Mbeya highlands. Here household decision makers may be tempted to neglect protective measures such as the use of ITNs for their children, owed to a rarer occurrence of malaria. This study shows a relevant increase in bed net ownership only for Mlowo, one of the lower altitude sites with a higher prevalence of malaria infection. The other three sites did not show a consistent change in bed net ownership. However, due to an incomplete adaptive immunity in this altogether hypoendemic situation and inbound mobility of potentially parasitaemic individuals from hyperendemic areas, the population of these hypoendemic regions remains at particular risk for severe courses of malaria.

The model for baseline malaria infection indicated that higher SES is protective against malaria infection. However, in the regression model on change in infection status, no conclusive effect of SES change on switching to malaria negative was found.

The crude average NDVI values at baseline were similar for the two low lying sites, and for the two higher lying sites respectively. The reduction of NDVI—and thus vegetation—between visit 1 and visit 2 likely indicates decreased rainfall for Igurusi and Mlowo, the sites with the largest absolute and relative reductions in malaria prevalence. Only Isongole, the site at highest altitude of 2040 m, showed an increase in NDVI; this site, however, contributed least to the overall reduction in malaria prevalence. However, when adjusting for site and household in the models on change in *P. falciparum* status (Table 5) this correlation between decreasing NDVI and increased odds of losing *P. falciparum* positivity could not be ascertained at the individual level.

The most important limitation of this study is the lack of a control group which would have enabled us to directly compare the impact of ACT treatment on malaria with that of SP treatment, the previous standard of care. Instead the analysis relies on a before/after comparison, which might have been confounded by other factors that changed between the two visits. In order to overcome this, the most important factors that were associated with malaria at baseline were identified, and an analysis was performed in order to examine whether their change could explain the observed drop in *P. falciparum* prevalence, which was not the case. Furthermore, to the knowledge of the authors, no additional systematic malaria control activities such as indoor residual spraying (IRS) were implemented in the region during the study period. Although other uncontrolled factors also might have played a role in this, the change in first-line treatment as corroborated through this study seems to sufficiently explain the observed change in *P. falciparum* prevalence.

A selection bias imposed by the exclusion of participants that had missed consecutive visits cannot be ruled out.

The validity of NDVI as a proxy indicator of humidity and, therefore, of favourable conditions for vector habitats is limited. NDVI has shown to correlate with rainfall only in defined lower ranges of annual rainfall. Mbeya region with about 800 mm annual rainfall is already exceeding the upper limit of linear correlation. In addition, many other factors such as lag time and fine scale variability over time were not considered [23, 24]. Another weakness are uncontrolled influences of urban areas on NDVI values, as sealed surfaces and construction activities disturb the correlation between NDVI and potential presence of vector habitat.

## Conclusions

The introduction of ACT in the Mbeya Region coincides with a relevant reduction in malaria prevalence within a very short period of time. Since this decrease is not explained by other factors included in the presented analyses, the investigators believe that it reflects the effect of switching to ACT as first-line malaria therapy. Lower prevalence of *P. falciparum* infection implies a lower transmission and, therefore, lower morbidity and mortality in vulnerable age groups, which comprise children and adolescents between 5 and 20 years of age. The study area is by now considered a low risk area for malaria transmission. At the same time the local health authorities have adopted an improved approach of antimicrobial stewardship with the introduction of RDTs and by reserving anti-malarial treatment for patients with a positive RDT result. In addition, this development improves the adequate use of antibiotics, which are not administered as a fixed combination with anti-malarials anymore. These are important prerequisites for avoiding development of resistances against first line anti-malarials currently in use.

## Abbreviations

ACT: artemisinin-based combination therapy
EMINI: Establishment of the infrastructure for the Evaluation and Monitoring of the Impact of New Interventions
HRP2: histidine-rich protein 2
IRS: indoor residual spraying
ITN: insecticide-treated net
MMRC: Mbeya Medical Research Center
NDVI: Normalized Difference Vegetation Index
RDT: rapid diagnostic testing
SES: socio-economic status
SP: sulfadoxine/pyrimethamine
